# Editorial: Machine Learning Methods for High-Level Cognitive Capabilities in Robotics

**DOI:** 10.3389/fnbot.2019.00083

**Published:** 2019-10-22

**Authors:** Tadahiro Taniguchi, Emre Ugur, Tetsuya Ogata, Takayuki Nagai, Yiannis Demiris

**Affiliations:** ^1^Department of Information Science and Engineering, Ritsumeikan University, Kyoto, Japan; ^2^Department of Computer Engineering, Boǧaziçi University, Istanbul, Turkey; ^3^Department of Intermedia Art and Science, School of Fundamental Science and Engineering, Waseda University, Tokyo, Japan; ^4^Department of Systems Innovation, Graduate School of Engineering Science, Osaka University, Osaka, Japan; ^5^Department of Electrical and Electronic Engineering, Imperial College London, London, United Kingdom

**Keywords:** machine leading, cognitive robotics, language acquisition, neural networks, cognitive architecture, probabilistic models, robot learning

## 1. Introduction

Adaptive learning and emergence of integrative cognitive system that involve not only low-level but also high-level cognitive capabilities are crucially important in robotics (Cangelosi et al., 2010; Cangelosi and Schlesinger, 2015; Ugur and Piater, 2015; Tani, 2016; Taniguchi et al., 2016, 2018). Recent advancement in machine learning methods, e.g., deep learning and hierarchical Bayesian modeling, enables us to develop cognitive systems that integrate multi-level sensory-motor and cognitive capabilities. Low-level cognitive capabilities includes sensory perception, physical control, and behavioral motion generation, while high-level cognitive capabilities include logical inference, planning, and language acquisition. To create robots that can deal with uncertainty in our daily environment, developing machine learning methods that can integrate low-level and high-level is essential. Following the successfully organized session “the Workshop on Machine Learning Methods for High-Level Cognitive Capabilities in Robotics 2016” held in IEEE-IROS 2016^1^, we organized this Research Topic. We aimed to publish original papers about the state-of-the-art machine learning methods that contribute to modeling sensory-motor and cognitive capabilities in robotics.

## 2. About the Research Topic

We are pleased to present 9 research articles, related to motor and behavior learning, concept formation, language acquisition, and cognitive architecture. In this section, we briefly introduce each paper.

First, three papers focused on action and behavior learning. Imitation learning is an important topic related to the integration of high-level and low-level cognitive capability because it enables a robot to acquire behavioral primitives from social interaction including observation of human behaviors. Nakajo et al. proposed a machine learning method for viewpoint transformation and action mapping using a neural network having encoder-decoder architecture, i.e., sequence to sequence. In imitation learning, demonstrator and imitator have different perspectives. The method deals with the problem and produced a successful result. Nakamura et al. proposed a new machine learning method called Gaussian process-hidden semi-Markov model (GP-HSMM). GP-HSMM can segment continuous motion trajectories without defining a parametric model for each primitive. That comprises Gaussian process, which is a regression method based on Bayesian non-parametric, and hidden semi-Markov model. This method enables a robot to find motion primitives from complex human motion in an imitation learning scenario. Manipulation using the left and right arms is an essential capability for a cognitive robot. Zhang et al. proposed a neural-dynamic based synchronous-optimization scheme manipulators. It was demonstrated that the method enables a robot to track complex paths.

Second, two papers focused on the relationship between action and object concept. Andries et al. proposes the formalism for defining and identifying affordance equivalence. The concept of affordance can be regarded as a relationship between an actor, an action performed by this actor, an object on which the action is performed, and the resulting effect. Learning affordance, i.e., inter-dependency between action and object concept, is an important topic in this field. Taniguchi et al. proposed a new active perception method based on multimodal hierarchical Dirichlet process, which is a hierarchical Bayesian model for multimodal object concept formation method. The important aspect of the approach is that the policy for active perception is derived based on the result of unsupervised learning without any manually designed label data and reward signals.

Third, three papers are related to language acquisition and concept formation. Hagiwara et al. proposed hierarchical spatial concept formation method based on hierarchical multimodal latent Dirichlet allocation (hMLDA). They demonstrated that a robot could form concept for places having hierarchical structure, e.g., “around a table” is a part of “dining room,” using hMLDA, and became able to understand utterances indicating places in a domestic environment given by a human user. Yamada et al. described representation learning method that enables a robot to understand not only action-related words, but also logical words, e.g., “or,” “and” and “not.” They introduced an neural network having an encoder-decoder architecture, and obtained successful and suggestive results. Taniguchi et al. proposed a new multimodal cross-situational learning method for language acquisition. A robot became able to estimate of each word in relation with modality via which each word is grounded.

The final paper presents a framework for cognitive architecture based on hierarchical Bayesian models. Nakamura et al. proposed Symbol Emergence in Robotics tool KIT (SERKET) that can integrate many cognitive modules developed using hierarchical Bayesian models, i.e., probabilistic generative models, effectively without re-implementation of each module. Integration of low-level and high-level cognitive capability and developing an integrative cognitive system requires researchers and developers to construct very complex software modules, and this is expected to cause practical problems. Serket can be regarded as a practical solution for the problem, and expected to push the research field forward.

## 3. Next Step

With the tremendous success of the past three Special issues of this Research Topic, we organized follow-up workshops^2^ and a Research Topic^3^. Two survey papers related to the series of workshops have already been published (Taniguchi et al., 2018; Tangiuchi et al., 2019). We will also organize a workshop with the special emphasis on deep probabilistic generative models^4^ We believe that in order to create an artificial cognitive system, i.e., a robot, it is important to integrate low-level and high-level cognitive capabilities based on machine learning-based methods. We hope that this special issue will contribute to accelerating the robotics and machine learning studies that aims to create human-like cognitive systems that can behave in our real-world environment in collaboration with people.

## Author Contributions

All authors listed have made a substantial, direct and intellectual contribution to the work, and approved it for publication.

### Conflict of Interest

The authors declare that the research was conducted in the absence of any commercial or financial relationships that could be construed as a potential conflict of interest.

## Footnotes

^1^The Workshop on Machine Learning Methods for High-Level Cognitive Capabilities in Robotics 2016: http://mlhlcr2016.tanichu.com/

^2^The 2nd Workshop on Machine Learning Methods for High-Level Cognitive Capabilities in Robotics 2017: http://mlhlcr2017.tanichu.com/. The Workshop on Language and Robotics: http://iros2018.emergent-symbol.systems/.

^3^Research Topic Language and Robotics: https://www.frontiersin.org/research-topics/8861/language-and-robotics.

^4^The Workshop on Deep Probabilistic Generative Models for Cognitive Architecture in Robotics 2019: https://sites.google.com/site/dpgmcar2019/.

## References

[B1] Cangelosi A. Metta G. Sagerer G. Nolfi S Nehaniv C. Fischer K. (2010). Integration of action and language knowledge: a roadmap for developmental robotics. IEEE Trans. Auton. Ment. Dev. 2, 167–195. 10.1109/TAMD.2010.2053034

[B2] Cangelosi A. Schlesinger M. (2015). Developmental Robotics: From Babies to Robots. Cambridge, MA: MIT Press.

[B3] Tangiuchi T. Mochihashi D. Nagai T. Uchida S. Inoue N. Kobayashi I. (2019). Survey on fron- tiers of language and robotics. Adv. Robot. 33,700–730. 10.1080/01691864.2019.1632223

[B4] Tani J. (2016). Exploring Robotic Minds: Actions, Symbols, and Consciousness as Self-Organizing Dynamic Phenomena. Oxford, UK: Oxford University Press.

[B5] Taniguchi T. Nagai T. Nakamura T. Iwahashi N. Ogata T. Asoh H. (2016). Symbol emergence in robotics: a survey. Adv. Robot. 30,706–728. 10.1080/01691864.2016.1164622

[B6] Taniguchi T. Ugur E. Hoffmann M. Jamone L. Nagai T. Rosman B. (2018). Symbol emergence in cognitive developmental systems: a survey. IEEE Trans. Cogn. Dev. Syst. 10.1109/TCDS.2018.2867772. [Epub ahead of print].

[B7] Ugur E. Piater J. (2015). “Bottom-up learning of object categories, action effects and logical rules: from continuous manipulative exploration to symbolic planning,” in IEEE International Conference on Robotics and Automation (ICRA) (Seattle, WA), 2627–2633.

